# TNM Staging of Pancreatic Neuroendocrine Tumors: An Observational Analysis and Comparison by Both AJCC and ENETS Systems From 1 Single Institution

**DOI:** 10.1097/MD.0000000000000660

**Published:** 2015-03-27

**Authors:** Min Yang, Lin Zeng, Yi Zhang, Wei-guo Wang, Li Wang, Neng-wen Ke, Xu-bao Liu, Bo-le Tian

**Affiliations:** From the Department of Hepatobiliopancreatic Surgery (MY, YZ, W-gW, LW, N-wK, X-bL, B-lT); and General Ward of Sports Medicine and Cardiopulmonary Rehabilitation (LZ), West China Hospital of Sichuan University, Chengdu, Sichuan, People's Republic of China.

## Abstract

We aimed to analyze the clinical characteristics and compare the surgical outcome of pancreatic neuroendocrine tumors (p-NETs) using the 2 tumor-node-metastasis (TNM) systems by both the *American Joint Committee on Cancer (AJCC) Staging Manual* (seventh edition) and the European Neuroendocrine Tumor Society (ENETS). Moreover, we sought to validate the prognostic value of the new AJCC criterion.

Data of 145 consecutive patients who were all surgically treated and histologically diagnosed as p-NETs from January 2002 to June 2013 in our single institution were retrospectively collected and analyzed.

The 5-year overall survival (OS) rates for AJCC classifications of stages I, II, III, and IV were 79.5%, 63.1%, 15.0%, and NA, respectively, (*P* < 0.005). As for the ENETS system, the OS rates at 5 years for stages I, II, III, and IV were 75.5%, 72.7%, 29.0%, and NA, respectively, (*P* < 0.005). Both criteria present no statistically notable difference between stage I and stage II (*P* > 0.05) but between stage I and stages III and IV (*P* < 0.05), as well as those between stage II and stages III and IV (*P* < 0.05). Difference between stage III and IV by ENETS was significant (*P* = 0.031), whereas that by the AJCC was not (*P* = 0.144). What's more, the *AJCC Staging Manual* (seventh edition) was statistically significant in both uni- and multivariate analyses by Cox regression (*P* < 0.005 and *P* = 0.025, respectively).

Our study indicated that the ENETS TNM staging system might be superior to the *AJCC Staging Manual* (seventh edition) for the clinical practice of p-NETs. Together with tumor grade and radical resection, the new AJCC system was also validated to be an independent predictor for p-NETs.

## INTRODUCTION

Pancreatic neuroendocrine tumors (p-NETs) are a group of heterogeneous neoplasm, which may derive not only from mature pancreatic endocrine cells, but also from pluripotent stem cells of the pancreas.^1^ With an obviously increasing incidence in the past 2 decades, p-NETs are still uncommon, accounting for <3% of all pancreatic tumors.^2,3^ It is a common practice to label p-NETs as functional if patients present the symptoms related to hormone overproduction, such as insulinoma, gastrinoma, and glucagonoma, and nonfunctional if they do not.^4^

Due to their rarity and heterogeneous behavior, the ability to stratify patients with p-NETs into groups for survival analysis has been challenging. Based on the clinicopathologic features of tumor, the classifications of p-NETs have experienced a long-time developing process.^5–8^ However, the more applicable classifications that were closely analogous with the tumor-node-metastasis (TNM) staging system used in other solid tumors were in urgent need. In 2006, 1 TNM staging system for p-NETs was firstly suggested and soon adopted by the European Neuroendocrine Tumor Society (ENETS), which simultaneously included a grading proposal for neuroendocrine tumors.^9^ In addition, the American Joint Committee on Cancer (AJCC) did not propose a specially available TNM staging system for p-NETs until the year 2010 (ie, the seventh edition), which was initially applied for the exocrine adenocarcinoma of pancreas.^10^

The definition of T stage, derived clinical stages, and original purpose of these 2 TNM systems differ greatly from each other (Table 1). Moreover, the TNM staging system of ENETS has provided great value for the treatments and prognostic stratifications of p-NETs, which has been already confirmed by some precious studies.^11–14^ On the contrary, the clinical and prognostic value of the new AJCC criteria has been seldom validated.^15,16^ In the present study, on the basis of the data of 145 consecutive patients in our single center for the past 11 years, we attempted to analyze the clinical characteristics and surgical outcome of p-NETs using the 2 TNM staging systems by both *AJCC Staging Manual* (seventh edition) and ENETS, emphasized on making a comparison with the survival differences among stages of both systems and identifying probably the more accurate and useful one for p-NETs. What's more, we would also validate the prognostic value of this new AJCC criterion.

**TABLE 1:**
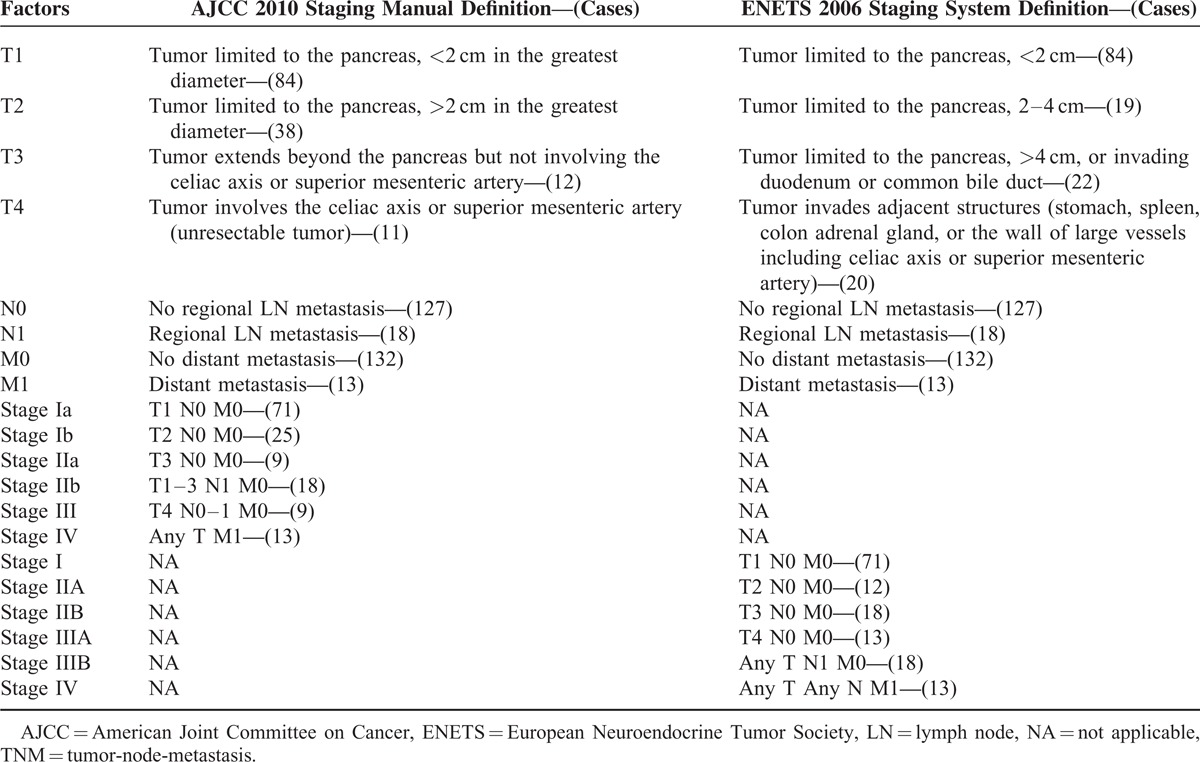
Original Definition and Present Analysis of the 2 TNM Staging Criteria

## MATERIALS AND METHODS

Clinical data of 145 consecutive patients who were all surgically treated and histologically diagnosed as p-NETs from January 2002 to June 2013 at West China Hospital of Sichuan University were retrospectively collected from their electronic or paper-based medical records. Patients with only clinical suspicion but not postoperatively pathologic confirmations of p-NETs were not enrolled in this study. All neoplasms were of pancreatic origin, and patients with tumors arising from the Vater ampulla, bile duct, or duodenum were excluded. All tumors were sporadic, and patients with hereditary syndrome, including 4 patients with multiple endocrine neoplasia type I, 2 with von Hippel–Lindau syndrome, and 1 with neurofibromatosis, were also excluded. The research was approved by the local ethics committee, and written consent was provided for patient information to be used for research purposes.

Our assessment systematically reviewed the data of eligible patients including patients’ demographics (sex and age at diagnosis), clinical manifestations (functional status), localization, size of the primary tumor, histopathologic diagnosis (lymph node involvement, vascular invasion, presence of metastasis, immunohistochemical staining, mitotic count, Ki-67-positive index, etc), surgical procedures and complications, total and postoperative hospital stays, and so on. The clinical TNM stages by *AJCC Staging Manual* (seventh edition) in 2010 and ENETS criteria in 2006 were both performed for all patients primarily referring to the results of preoperative imaging studies, intraoperative surgical findings, and postoperative pathologic analysis. The grading proposal by ENETS in 2006 was also applied wherever possible to analyze the histopathologic features of p-NETs in our research, which was mainly based on mitotic count and Ki-67-positive proliferative index. For better understanding, we accordingly defined all subjects as follows: well differentiated (G1: mitotic count <2/10 high-power fields [HPF], Ki-67 ≤ 2%), moderately differentiated (G2: mitotic count 2–20/10 HPF, Ki-67 3%–20%), and poorly differentiated (G3: mitotic count >20/10 HPF, Ki-67 > 20%).

Follow-up was done by telephone, office visit, and outpatient clinic or physical examination from February to June, 2014, giving a potential follow-up time from 6.21 to 136.02 months and a median of 72.85 months. Fourteen patients were lost to follow-up and were excluded from the survival analysis. Deaths classified as not being related to p-NETs were not enrolled in this study. Overall survival (OS) was calculated as the number of months from the date of operation to the day of last contact or time of death.

Distribution of continuous variables was reported as mean ± standard error of the mean unless otherwise indicated. Categorical variables were presented as numbers and their frequencies as proportions (percentage). Kaplan–Meier estimates of survival curves were plotted, and survival differences between stages were compared using the log-rank test. Univariate and multivariate analyses were used to explore the effects of several prognostic factors by the Cox regression proportional hazards model. Statistical significance was considered when *P* value of 2 sides was <0.05. Statistical analyses were performed using IBM SPSS17.0 statistical software of International business machines co., LTD in Beijing, China.

## RESULTS

### Patient Characteristics

There were 145 patients in total with histologically diagnosed p-NETs after resection from January 2002 to June 2013 at the West China Hospital of Sichuan University, whose features were mostly summarized in Table 2. The present cohort comprised 60 males and 85 females, with a male-to-female ratio of 0.7:1. Median age at initial diagnosis was 46 years, with a mean of 46.16 ± 13.68 (range 14–77 years). Tumor diameters ranged from 0.3 to 12 cm, with an average of 2.95 ± 2.57 cm and a median of 2 cm. The majority of p-NETs in this cohort (95, 65.5%) were associated with a hormonal syndrome (ie, functioning), in which 81 patients were clinically and pathologically diagnosed as insulinoma (55.7%), whereas a total of 50 patients (34.5%) did not present the symptoms related to hormone overproduction (ie, nonfunctioning). Sixty tumors were located in the head and uncinate of pancreas, 85 in the body and tail. In terms of the tumor grading, well, moderately, and poorly differentiated tumors were diagnosed in 66, 41, and 13 patients, respectively. Radical resection was performed on 128 patients, whereas 17 patients only underwent palliative or explorative operations. When the follow-up ended in June 2014, 94 patients were alive and 37 patients were dead, with a death rate of 28.2%. Fourteen patients were lost to contact and were then excluded from the survival analysis.

**TABLE 2:**
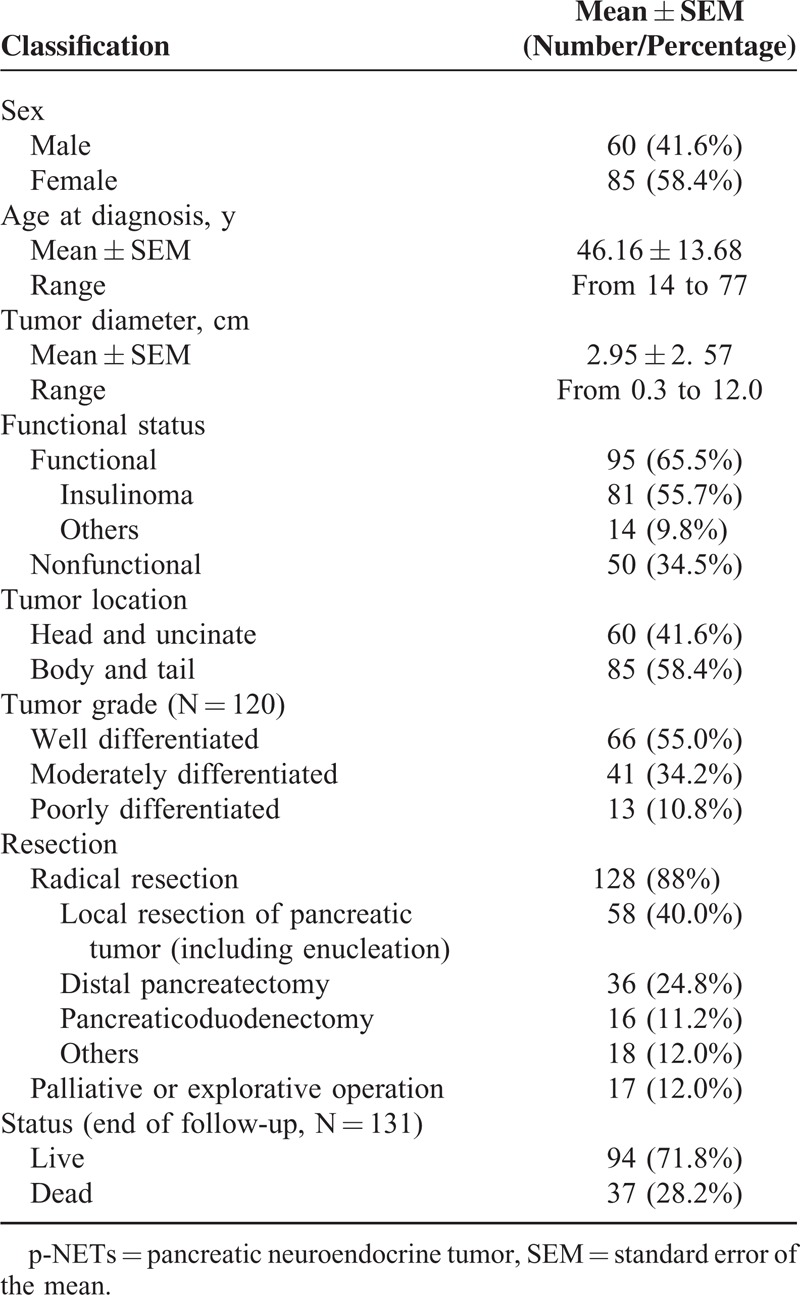
Clinical Features of the Entire Cohort With p-NETs

### Stages and Survivals by AJCC and ENETS

A TNM stage was assigned for each patient according to the new *AJCC Staging Manual* (seventh edition in 2010) and ENETS 2006 staging systems (Table 1). There were 84, 38, 12, and 11 patients from stages T1 to T4, respectively, by the AJCC criteria, and 84, 19, 22, and 20 cases, respectively, by the ENETS criteria. Eighteen patients were pathologically confirmed to have invasions of lymph node, whereas 13 present distant metastases. In terms of the clinical stages, stages I, II, III, and IV by AJCC criteria were defined in 96, 27, 9, and 13 patients, respectively, whereas those of ENETS criteria were classified in 71, 30, 31, and 13 patients, respectively.

**FIGURE 1:**
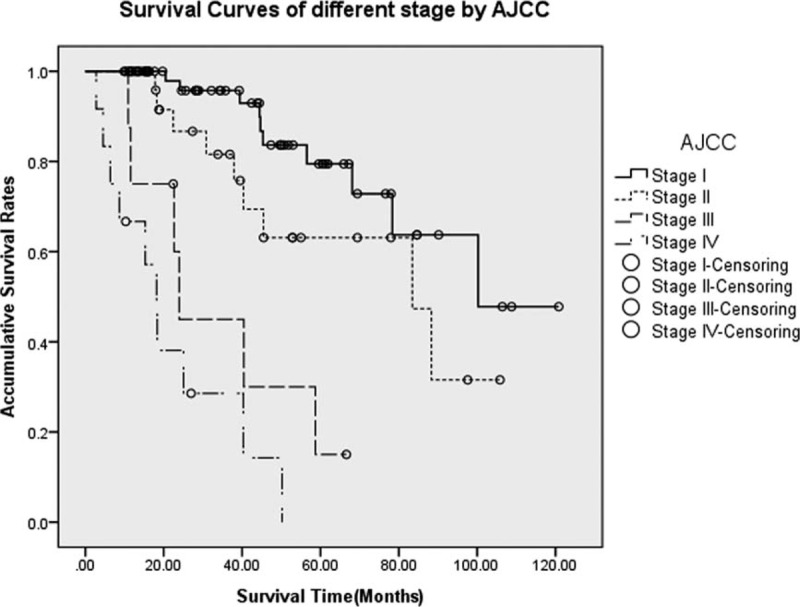
Survivals for p-NETs in different stages by the *AJCC Staging Manual (seventh edition)*. Differences were statistically significant when comparing stage I with stages III and IV (*P* < 0.005 and *P* < 0.005, respectively), as well as stage II with stages III and IV (*P* = 0.018 and *P* < 0.005, respectively), whereas those of stage I with stage II and stage III with stage IV did not present notable differences (*P* = 0.085 and *P* = 0.144, respectively). AJCC = American Joint Committee on Cancer, p-NETs = pancreatic neuroendocrine tumor.

The 5-year OS rates for AJCC classification stages I, II, III, and IV were 79.5%, 63.1%, 15.0%, and NA (could not be calculated), respectively (*P* < 0.005, Figure 1); median survival time (MST) for each stage was 100.2, 88.4, 23.9, and 18.2 months, respectively. The survival differences were statistically significant when comparing stage I with stages III and IV (*P* < 0.005 and *P* < 0.005, respectively), as well as stage II with stages III and IV (*P* = 0.018 and *P* < 0.005, respectively), whereas comparisons of stage I with II and stage III with IV did not present notable differences (*P* = 0.085 and *P* = 0.144, respectively).

**FIGURE 2:**
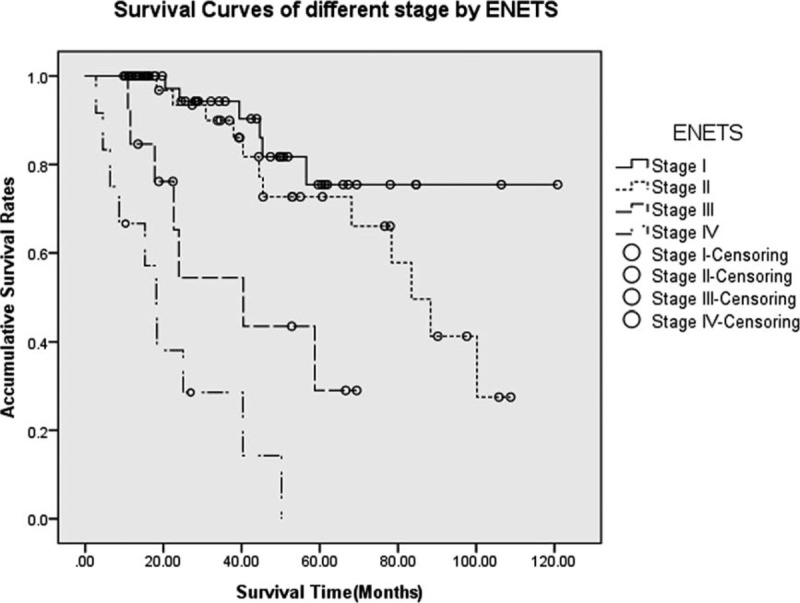
Survivals for p-NETs in different stages by the ENETS classification. Differences between stage I and stages III and IV were statistically significant (*P* = 0.001 and *P* < 0.005, respectively), as well as those between stage II and stages III and IV (*P* = 0.009 and *P* < 0.005, respectively). Meanwhile, there was no statistically obvious difference between stage I and stage II (*P* = 0.207), whereas notable difference was detected between stages III and IV (*P* = 0.031). ENETS = European Neuroendocrine Tumor Society, p-NETs = pancreatic neuroendocrine tumor.

As for the ENETS system, the 5-year OS rates for stages I, II, III, and IV were 75.5%, 72.7%, 29.0%, and NA (could not be calculated), respectively (*P* < 0.005, Figure 2); the MST for each stage was NA, 83.4, 43.4, and 18.2 months, respectively. Differences of survival between stage I and stages III and IV were statistically significant (*P* = 0.001 and *P* < 0.005, respectively), as well as those between stage II and stages III and IV (*P* = 0.009 and *P* < 0.005, respectively), whereas no obvious difference was detected between those of stage I and stage II (*P* = 0.207). However, there was statistically notable difference between the survival rates of stage III and IV (*P* = 0.031).

### Survivals by Grades and Resections

**FIGURE 3:**
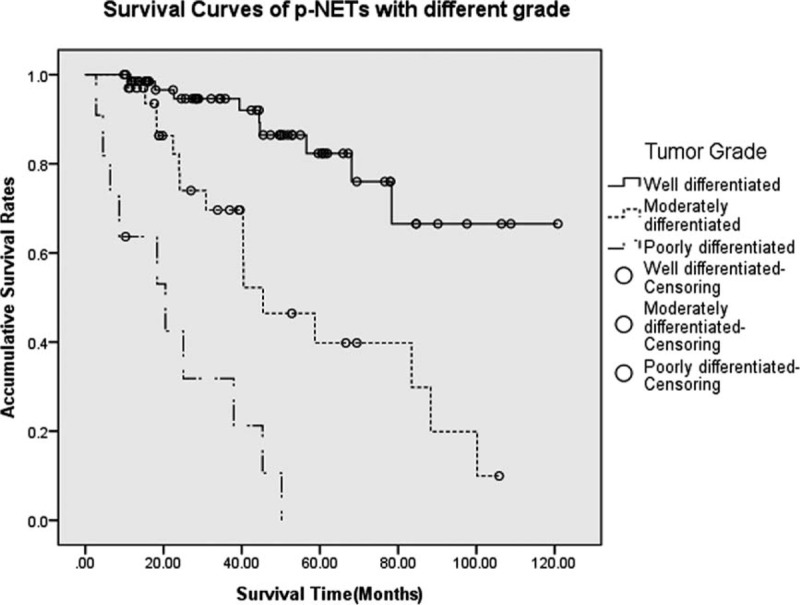
Survivals for p-NETs with different grades. Differences between well and moderately and poorly differentiated tumors were statistically significant (*P* < 0.005 and *P* < 0.005, respectively), as well as those between moderately and poorly differentiated ones (*P* < 0.005). p-NETs = pancreatic neuroendocrine tumor.

Histopathologic grade was assigned for 3 groups based on the available information from a total of 120 patients (82.8%), with a distribution of 66, 41, and 13 patients, respectively. The 5-year OS rates for well and moderately, well and poorly differentiated tumors were 82.3%, 39.8%, and NA, respectively, with a MST of NA, 45.4, and 20.5 months, respectively. OS varied markedly based on tumor grade (Figure 3), whose differences between well and moderately, well and poorly differentiated tumors were statistically significant (*P* < 0.005 and *P* < 0.005, respectively), as well as those between moderately and poorly differentiated ones (*P* < 0.005).

**FIGURE 4:**
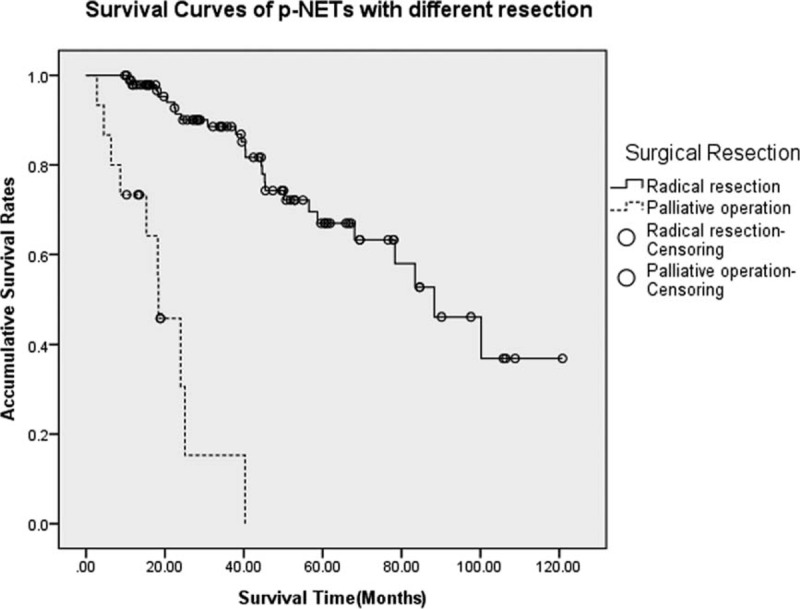
Survivals for p-NETs with different resections. Patients undergoing radical resections present a statistically better survival than those who only underwent palliative or explorative operations (*P* < 0.005). p-NETs = pancreatic neuroendocrine tumor.

All patients in this study received surgical treatments so that the diagnosis of p-NETs could be histologically confirmed from the resected tissues or biopsies. There were totally 128 patients undergoing radical resections (88%), including local resection of pancreatic tumor (40.0%), distal pancreatectomy (24.8%), pancreaticoduodenectomy (11.2%), and others (12.0%), whereas palliative or explorative operations were performed only on 17 patients (12.0%). With an MST of 88.3 and 18.4 months, respectively, patients undergoing radical resections present a statistically better survival than those who did not (OS at 5 years were 67.1% and NA, respectively) (*P* < 0.005, Figure 4).

### Analysis of Prognostic Factors

Cox regression was performed analyzing the probable predictors of p-NETs (Table 3). MST did not significantly differ between the sexes (58.7 vs 88.3 months, *P* = 0.086), as well as that of tumor location (68.1 vs 83.4 months, *P* = 0.214). Patients <46 years obtained a statistically longer MST than those >46 years (88.3 vs 52.1 months, *P* = 0.045), whereas that of patients with tumor size >2 and <2 cm present no notable difference (68.1 vs 88.3 months, *P* = 0.136). Patients with functional tumors had a MST of 80.2 months, compared statistically 45.3 months of those with nonfunctional tumors (*P* = 0.005). Moreover, tumor grade (well vs poorly and moderately), radical resection (yes vs no), and tumor stage by AJCC (stages I and II vs stages III and IV) were also statistically significant in the univariate analysis (*P* < 0.005, *P* < 0.005, and *P* < 0.005; respectively). Bringing these 5 significant factors directly into the Cox multivariate regression proportional hazards model, we concluded tumor stage by AJCC and tumor grade and radical resection as independent predictors for p-NETs (relative death risk [RDR] = 1.731, 95% confidence interval [CI] 1.072–2.796, *P* = 0.025; RDR = 0.606, 95% CI 0.372–0.987, *P* = 0.044; RDR = 0.501, 95% CI 0.299–0.839, *P* = 0.009; respectively).

**TABLE 3:**
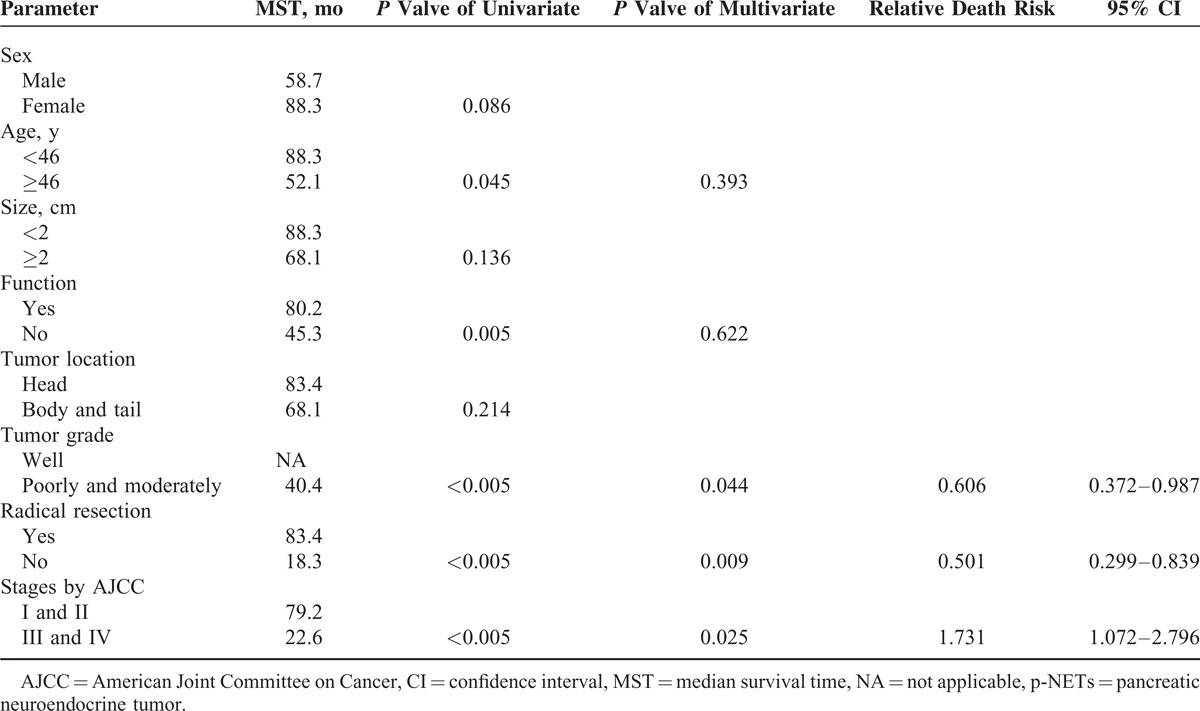
Uni- and Multivariate Analysis of Predictors for p-NETs by Cox Regression

## DISCUSSION

Due to the overall rare and heterogeneous behavior with indolent malignancy, p-NETs have not been well studied as pancreatic adenocarcinoma.^17^ In 2006, Rindi et al^9^ first proposed a 4-stage TNM classification for gastrointestinal and pancreatic NETs, which has subsequently been adopted by ENETS and evaluated.^11–14^ On the contrary, the TNM staging system by the *AJCC Staging Manual* (seventh edition) in 2002 (ie, the sixth edition) excludes p-NETs as usual when staging pancreatic tumors,^18^ which was nevertheless validated in 2007 by Bilimoria et al^19^ that this staging manual could also provide good prognostic survival discriminations between stage groups for resected patients, and that it could effectively stratify patients with p-NETs. This might provide theoretical basis and practical value for the development of this new criterion. Subsequently, in 2010, the *AJCC Staging Manual* (seventh edition) first introduced its TNM staging classifications to p-NETs, which derived from the staging algorithm for pancreatic exocrine adenocarcinomas.^10^ This represented an important step toward adopting a uniform p-NETs staging system with widespread acceptance and is now endorsed by both the International Union for Cancer Control^20^ and the World Health Organization.^21^ Therefore, the presence of these 2 TNM staging systems for p-NETs by both ENETS and *AJCC Staging Manual* (seventh edition) might raise clinical concerns of potential confusions in patient management.^22,23^ What's more, due to the more indolent biologic behavior, p-NETs are regarded to have better long-term survival rates than pancreatic exocrine tumors.^24,25^ Therefore, use of a common staging system for the 2 different disease processes, although convenient, might be oversimplified or improper. In 2011, based on 11 years, data of 425 patients with p-NETs from their single institution, Strosberg et al^15^ first succeeded in evaluating the clinical value of this *AJCC Staging Manual* (seventh edition) for p-NETs, in which they concluded that the TNM classifications by the new AJCC criterion was prognostic for OS rates of p-NETs and that it could be adopted in clinical practice. This result was once again validated in its subsequent analysis for surgically resected patients with p-NETs.^26^ In this research, we analyzed the clinical characteristics and surgical outcome of p-NETs using these 2 TNM staging systems for p-NETs by both ENETS and *AJCC Staging Manual* (seventh edition), made a simple comparison to find probably the more practical one for survival analysis of p-NETs. We also tried to validate the prognostic value of this new *AJCC Staging Manual*.

The stage-stratified 5-year OS rates of stages I, II, III, and IV by this new AJCC classification derived from our single institutional cohort were 79.5%, 63.1%, 15.0%, and NA, respectively, which were markedly lower than what Strosberg et al^15^ have reported (92%, 84%, 81%, and 57%, respectively, *P* < 0.001). This was probably due to the different features of the population studied and the different designs of each research. Specifically, compared with theirs, we had a relatively smaller number of sample size and a shorter follow-up time. Also, patients in our research showed a higher mortality rate, especially in the early period, many study objects were either lost to follow-up or dead. Nevertheless, in accordance with their study results, we also investigated that the survival differences by the new *AJCC Staging Manual* were statistically significant when comparing stage I or II with stage III or IV (*P* < 0.005), whereas comparisons of stage I with stage II or stage III with IV both present no statistically notable differences (*P* > 0.05), which might probably be a potential limitation of this new classification.

In addition, on the basis of 17 years, data of 1072 patients with p-NETs who had previously underwent surgical treatment in a large international database, Rindi et al^27^ in 2012 made a head-to-head comparison for the first time between the *AJCC Staging Manual* (seventh edition) and the ENETS classification. They reported that the ENETS TNM staging system could perfectly allocate patients into 4 statistically different and equally populated risk groups (*P* < 0.001), whereas the AJCC criterion only compressed the disease into 3 differently populated classes (*P* < 0.001). Further investigations prompted their eventual conclusions that, though both TNM staging systems were independent predictors of survival for p-NETs, the ENETS criterion was superior to the *AJCC Staging Manual* (seventh edition). As shown in Table 1, the definition of T stage and derived clinical stages of these 2 TNM systems differ greatly from each other. In our study, there were 84, 38, 12, and 11 patients from stages T1 to T4, respectively, by the new *AJCC Staging Manual*, and 84, 19, 22, and 20, respectively, by the ENETS staging system. So, stages I to IV by AJCC were defined in 96, 27, 9, and 13 patients, respectively, whereas those of ENETS were classified in 71, 30, 31, and 13 patients, respectively. Then, when performing the survival analysis and comparison, we worked out that both systems present no statistically notable difference between stage I and stage II (*P* > 0.05) but between stage I and stages III and IV (*P* < 0.05), as well as between stage II and stages III and IV (*P* < 0.05). However, difference between stages III and IV by the ENETS 2006 TNM staging system was statistically significant (*P* = 0.031), which might be an advantage over the *AJCC Staging Manual* (seventh edition) (*P* = 0.144).

In the present study, we also validated the prognostic value of the TNM systems by the *AJCC Staging Manual (seventh edition)*, which was both statistically significant in uni- and multivariate analyses by Cox regression (*P* < 0.005 and *P* = 0.025, respectively). Besides, our institutional retrospective analysis also confirmed that tumor grade and radical resection were critically important and independent predictors for patients with surgically treated p-NETs (*P* = 0.044 and *P* = 0.009, respectively). Patients in early stage by AJCC with well differentiated p-NETs and those undergoing radical resection often present a better survival, which was in agreement with previous reports.^15,16,27^ Moreover, the prognostic value of tumor grading by ENETS also showed the other advantage over the *AJCC Staging Manual (seventh edition)* criterion.

The major limitation of our study is its nature of retrospective investigation, which implies some potential degree of variation in collecting relevant data, such as the tumor histopathologic features and patients’ follow-up. Secondly, any in-depth or further research for different stages and factors is still needed to validate their prognostic valve. What's more, collection of relevant data in a large prospective series with uniform protocols for data entry is also needed to confirm our results.

In conclusion, the TNM staging systems by both the *AJCC Staging Manual (seventh edition)* and the ENETS classification are applicable for p-NETs. Considering the advantages of staging and grading proposal by ENETS, our study also indicated that the ENETS system might be superior to the *AJCC Staging Manual (seventh edition)* criterion and supported its extensive use in current clinical practice of p-NETs. Moreover, apart from tumor grade and radical resection, the *AJCC Staging Manual (seventh edition)* was validated as well to own its prognostic value for p-NETs.
