# TNM Staging of Pancreatic Neuroendocrine Tumors: An Observational Analysis and Comparison by Both AJCC and ENETS Systems From 1 Single Institution: Erratum

**DOI:** 10.1097/01.md.0000482828.40339.51

**Published:** 2016-05-06

In the article “TNM Staging of Pancreatic Neuroendocrine Tumors: An Observational Analysis and Comparison by Both AJCC and ENETS Systems From 1 Single Institution”,^1^ which appeared in Volume 94, Issue 12 of *Medicine*, Drs. Yang, Zeng, and Zhang contributed equally to the article and share first coauthorship. The article has since been corrected online.
